# Endocervical Adenocarcinoma Showing Microcystic, Elongated, and Fragmented (MELF) Pattern of Stromal Invasion: A Single-Institutional Analysis of 10 Cases with Comprehensive Clinicopathological Analyses and Ki-67 Immunostaining

**DOI:** 10.3390/biomedicines11113026

**Published:** 2023-11-11

**Authors:** Hyunsik Bae, Hyun-Soo Kim

**Affiliations:** Department of Pathology and Translational Genomics, Samsung Medical Center, Sungkyunkwan University School of Medicine, Seoul 06351, Republic of Korea; hyunsik.bae@samsung.com

**Keywords:** uterus, mesonephric-like adenocarcinoma, targeted sequencing, immunohistochemistry, programmed cell death-ligand 1, mismatch repair

## Abstract

Microcystic, elongated, and fragmented (MELF) pattern of invasion has seldom been documented in endocervical adenocarcinoma (EAC). The aim of this study was to analyze the clinicopathological characteristics of EAC showing MELF pattern. We collected the clinicopathological information of 10 cases of EAC with the MELF pattern and conducted polymer-based immunostaining for Ki-67 (dilution 1:200, clone MIB-1) on these cases. Ki-67 expression was assessed using the average estimation within the hotspot method. All tumors were human papillomavirus-associated EAC with Silva pattern C. All except one tumor exceeded 3 cm in size. Five tumors involved the entire thickness of the cervical stroma, and four tumors extended into the parametrium. Lymphovascular space invasion was identified in six cases. Two patients developed metastatic recurrences in the para-aortic lymph nodes and lungs, respectively. The MELF area showed significantly lower Ki-67 labelling index than that of a conventional tumor area. We confirmed our previous observation that the MELF area displayed lower proliferative activity than the conventional tumor area of EAC. We also demonstrated that patients with EAC showing MELF pattern had several adverse clinicopathological characteristics reflecting aggressive behavior. On the other hand, since the frequencies of post-operative recurrence and disease-related mortality that occurred during the follow-up period were relatively low, further investigations are warranted to clarify the prognostic value of MELF pattern in EAC patients.

## 1. Introduction

Endocervical adenocarcinoma (EAC) is the fourth most commonly diagnosed carcinoma in women worldwide. EAC accounts for 20–25% of all cervical carcinoma cases [1,2]. In recent years, the incidence of EAC has steadily increased, particularly in patients 30 or older [3]. EAC is a histologically diverse group of tumors with various etiologies and molecular drivers [4]. Most EACs are related to infection with high-risk oncogenic human papillomavirus (HPV), with HPV genotypes 18, 16, and 45, accounting for >90% of the cases [5]. Unlike cervical squamous cell carcinoma, approximately 15–20% of EACs are not associated with high-risk HPV infection and harbor distinct molecular alterations.

EACs have traditionally been diagnosed based on morphology, primarily on tumor architecture and the presence of intracytoplasmic mucin [3]. The diagnoses of many histological types included in the 2014 World Health Organization (WHO) Classification of Tumors of Female Reproductive organs were poorly reproducible [6], with very little clinical relevance. The International Endocervical Adenocarcinoma Criteria and Classification (IECC) divided EACs into HPV-associated (HPVA) and HPV-independent (HPVI) tumors, based on the presence of HPV infection-related histological features: easily identifiable apical mitotic figures and basal apoptotic bodies [4]. Compared with the 2014 WHO Classification, the 2018 IECC has shown superior interobserver agreement among gynecological pathologists and a highly significant correlation with HPV status, suggesting it is a more clinically valuable system for classifying EAC [3]. The most common type is usual-type HPVA EAC, representing 75% of all EAC cases [6]. Gastric-type HPVI EAC is the second most common type of EAC [4]. HPVI EAC is known to exhibit more aggressive clinical behavior and worse prognosis than HPVA EAC [7]. The varied morphology of these tumors results in diverse problems in differential diagnosis and therapeutic management.

Microcystic, elongated, and fragmented (MELF) pattern is a distinctive pattern of myometrial invasion occasionally observed in the invasive front of endometrial endometrioid carcinoma (EEC) [8]. Lee et al. [9] reviewed 93 cases of myoinvasive EEC to determine whether any histological features, other than those traditional clinicopathological parameters, might predict disease recurrence. They mentioned that diffuse myometrial invasion and associated inflammatory response were associated with an increased risk for recurrence of EEC. Murray et al. [10] first coined the term MELF for distinctive morphological changes observed in the invasive front of EEC, characterized by elongated microcysts lined by flattened epithelium, fragmented small cellular clusters, and single cells detached from typical neoplastic glands. They reported that these epithelial changes were associated with fibromyxoid stromal reaction and varying degrees of inflammatory infiltrates [11]. The presence of MELF pattern has been reported to be associated with lymphovascular space invasion (LVSI), regional lymph node metastasis (LNM), and deep myometrial invasion in EEC patients [12,13].

Despite being known as a poor pathological parameter, MELF pattern of stromal invasion in EAC has been studied very rarely. There has been the only multi-institutional study reporting the clinicopathological significance and prognostic value of MELF pattern in EAC [14]. Although MELF pattern is correlated with several aggressive pathological characteristics, it is not independently associated with the survival of EAC patients [14]. We recently encountered a case of usual-type HPVA EAC with MELF pattern. In this case, we observed that the MELF area displayed a very low proliferative activity compared to those of a conventional tumor area [15]. In this study, we comprehensively investigated the clinicopathological characteristics of 10 patients with EAC showing MELF pattern. We also conducted immunostaining for Ki-67 to examine whether there is a significant difference in proliferative activity between the MELF and conventional tumor areas.

## 2. Materials and Methods

### 2.1. Case Selection and Clinicopathological Data Collection

**Figure 1:**
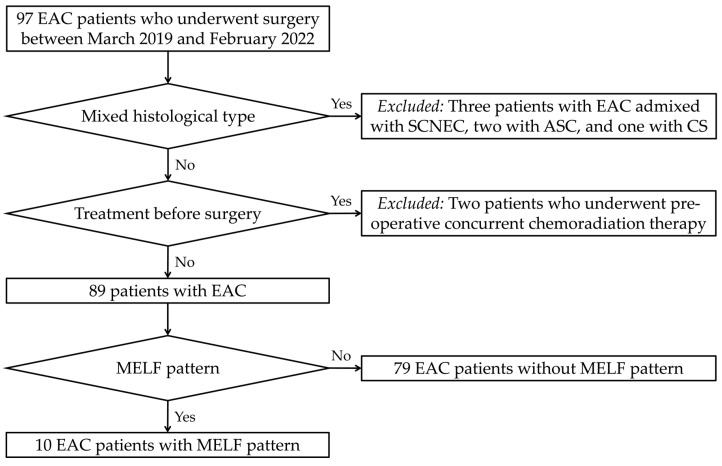
Flowchart of patient selection process. Among 97 patients with primary EAC, 8 were excluded from this study due to either mixed histology (three with small cell neuroendocrine carcinoma [SCNEC], two with adenosquamous carcinoma [ASC], and one with carcinosarcoma [CS]) or pre-operative treatment (two patients). We included 89 patients who underwent surgery for EAC in our institution. Slide review reveals that 10 cases of EAC displayed MELF pattern.

**Figure 2:**
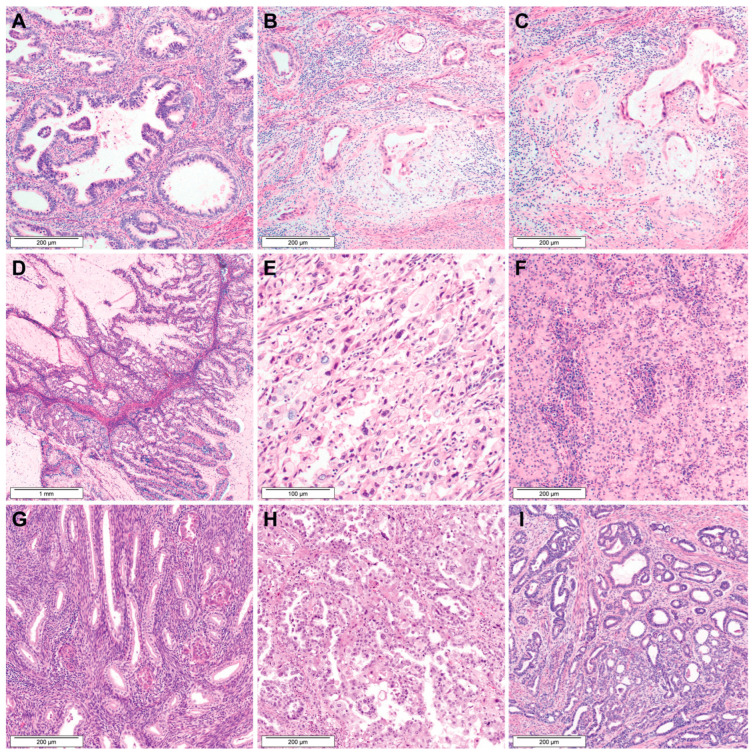
Histological types of EAC. (**A**) Usual type. (**B**,**C**) Usual type with MELF pattern. (**D**) Mucinous intestinal type. (**E**) Signet-ring cell type. (**F**) Invasive stratified mucin-producing type. (**G**) Gastric type. (**H**) Clear cell type. (**I**) Mesonephric type.

Figure 1 shows the flowchart of the participant selection process. Between March 2019 and February 2022, we studied 97 patients who underwent surgery for primary EAC from the institutional databases. Two board-certified pathologists reviewed all available hematoxylin and eosin-stained slides to confirm the diagnosis and to examine the presence of MELF pattern. Representative photomicrographs showing the histological features of EAC types are illustrated in Figure 2. We excluded six patients whose tumors exhibited mixed histological types, including EAC mixed with small cell neuroendocrine carcinoma (three cases), adenosquamous carcinomas (two cases), and carcinosarcoma (one case). Two patients who underwent pre-operative concurrent chemoradiation therapy were also excluded. Consequently, 89 patients with primary EAC were included in this study. Among these, MELF pattern was identified in 10 cases. We reviewed the electronic medical records and pathology reports to collect the following clinicopathological information: age at diagnosis; initial clinical presentation; imaging finding; surgical treatment; post-operative treatment and recurrence; disease-free survival (DFS); survival status; overall survival (OS); histological type; greatest dimension and invasion depth of tumor; Silva pattern of invasion; LVSI; extension to the uterine corpus and serosa, parametrium, and vagina; LNM; and initial stage.

### 2.2. Immunohistochemical Staining

Immunostaining for Ki-67 was performed using whole-tissue sections as described previously [16,17,18,19,20,21,22,23,24]. For each case, the most representative slide containing >80% of viable tumor tissue was chosen for immunostaining. Two patients did not provide sufficient tumor tissue for immunostaining. In brief, 4 µm thick, formalin-fixed, paraffin-embedded tissue sections were deparaffinized and rehydrated using a xylene and alcohol solution. IHC was performed using the Bond Polymer Intense Detection System (Leica Biosystems, Buffalo Grove, IL, USA) [25]. After antigen retrieval, endogenous peroxidases were quenched with hydrogen peroxide. Next, the sections were incubated with primary antibodies against Ki-67 (1:200, clone MIB-1, Agilent Technologies, Santa Clara, CA, USA). A biotin-free polymeric horseradish peroxidase-linker antibody conjugate system was used with a Bond-max automated immunostainer (Leica Biosystems, Buffalo Grove, IL, USA). After chromogenic visualization, the sections were counterstained with hematoxylin. Appropriate controls were stained concurrently. The positive controls included primary endometrial serous carcinoma. The negative control was prepared by substituting non-immune serum for primary antibodies, resulting in no detectable staining.

The Ki-67 labelling index was assessed via visual examination of the immunostained slides using a light microscope (BX43 Microscope, Olympus, Tokyo, Japan). The percentage of positive Ki-67 expression was assessed using the average estimation within the hotspot method as described previously [26]. A hotspot was defined as the area with the highest density of Ki-67-positive tumor cells, identified at 100× magnification. Within this area, we assessed the proportion of Ki-67-positive tumor cells in relation to all the tumor cells at five high-power fields (400× magnification) covering the different areas of the tumor. For each case, the mean percentage of Ki-67-positive tumor cells per high-power field was then calculated.

### 2.3. Statistical Analysis

The independent two-sample *t*-test was used to compare the Ki-67 labelling indices between the MELF and conventional tumor areas. All statistical analyses were performed using IBM SPSS Statistics for Windows, version 23.0 (IBM Corporation, Armonk, NY, USA). Statistical significance was set at *p* value < 0.05.

## 3. Results

### 3.1. Clinical Characteristics

Table 1 summarizes the clinical characteristics of 10 patients with EAC showing MELF pattern. The patients’ ages ranged from 27 to 77 years (median = 47 years; mean = 51.3 years). Three patients presented with vaginal bleeding, and seven patients had abnormal cytological results, including endocervical adenocarcinoma (3/7); atypical glandular cells, not otherwise specified (2/7); high-grade squamous intraepithelial lesion (HSIL; 1/7); and atypical squamous cells, which cannot exclude HSIL (1/7). Pre-operative imaging findings were available for all except one patient. Eight of the nine tumors presented as a cervical mass, whereas the remaining one appeared to be an endometrial mass involving the lower uterine segment. The mean size of the tumors was 4.1 cm (median = 3.8 cm; range = 2.9–7.0 cm). Parametrial extension was suspected in two patients. None of the examined patients had nodal or distant metastasis. All patients underwent radical (8/10) or total (2/10) hysterectomy. Six patients underwent bilateral salpingo-oophorectomy, while three patients under 40 years of age underwent bilateral salpingectomy only. Pelvic and para-aortic lymph node dissections were conducted in nine and one patients, respectively. Six and three patients received post-operative adjuvant radiation therapy and concurrent chemoradiation therapy (CCRT), respectively. Two patients developed post-operative tumor recurrence in the para-aortic lymph nodes and lungs, respectively. Post-operative follow-up information was available for all except one patient, who was lost to follow-up after detection of para-aortic lymph node recurrence. Mean DFS and OS were 28.3 and 36.3 months, respectively (median = 19.5 and 25 months, respectively). One patient who experienced recurrent lung metastasis underwent metastasectomy and chemotherapy but died of disease 93 months after surgery.

### 3.2. Histological Features

**Figure 3:**
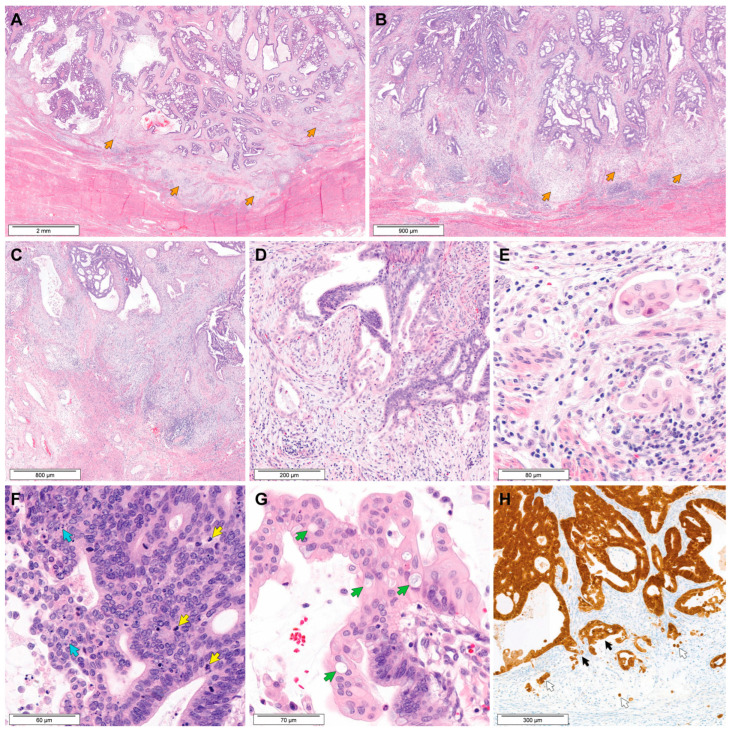
Histological features of MELF pattern in EAC. (**A**,**B**) Compared with the conventional tumor area showing well-developed, complex glandular architecture (upper one-third), the MELF area (orange arrows) displays a strikingly different structure. Note a poorly circumscribed, lobulated tumor border along the invasive front, with neoplastic glands not apparent at low-power magnification. (**C**) In the MELF area, the neoplastic glands seem to be variably collapsed or absent, and the stroma exhibits intense inflammatory reactions. (**D**) The lining epithelium of distorted or ruptured glands show a squamoid or histiocytoid appearance. A fibromyxoid response in the periglandular stroma is associated with mixed inflammatory infiltrates. (**E**) Some tumor cells scattered singly or forming small clusters resemble histiocytes. (**F**) Usual-type EAC characteristically shows human papillomavirus-infection-related histology, including apical mitotic figures (yellow arrows) and basal apoptotic bodies (blue arrows). Most of the tumor cells exhibit nuclear hyperchromasia and pleomorphism (inset). (**G**) Compared with a conventional tumor area, the tumor cells in the MELF area are round or polygonal with more abundant cytoplasm, resulting in a lower nuclear-to-cytoplasmic ratio. They exhibit smaller nuclei with mild pleomorphism, larger amount of eosinophilic cytoplasm, and intracytoplasmic vacuoles (green arrows). (**H**) Immunostaining reveals block p16 positivity in both the MELF (white arrows) and conventional invasion (black arrows) areas. Staining method: (**A**–**G**), hematoxylin and eosin staining; (**H**), immunohistochemical staining. Original magnification: (**A**,**B**), 20×; (**C**), 40×; (**D**), 100×; (**E**–**G**), 400×; (**H**), 100×.

Figure 3 demonstrates typical histological features of MELF pattern of stromal invasion in EAC. MELF pattern was evident at the invasive tumor front. Striking stromal desmoplasia was accompanied with myxoinflammatory responses and obscured the neoplastic glands (Figure 3A,B). This distinctive pattern of stromal invasion resulted in a poorly circumscribed, lobulated tumor border along the invasive front, with neoplastic glands not apparent at low-power magnification (Figure 3C). Scattered irregular-shaped or ruptured neoplastic glands were lined with degenerated or attenuated epithelial cells. Some of these glands had a single layer of epithelial lining showing polygonal contour and abundant clear-to-eosinophilic cytoplasm (Figure 3D). The tumor cells formed small clusters or were scattered singly in myxoinflammatory stroma. They closely resembled histiocytes (Figure 3E). In some areas, fragmented glandular epithelial cells had a squamoid appearance and formed microcysts. Cytologically, the tumor cells in the MELF area were morphologically different from the conventional usual-type EAC cells displaying apical mitotic figures and basal apoptotic bodies (Figure 3F). The tumor cells in the MELF area possessed smaller nuclei with irregular membranes, a larger amount of eosinophilic cytoplasm, more intracytoplasmic vacuoles, and a lower nuclear-to-cytoplasmic ratio than those in the conventional tumor area (Figure 3G). They exhibited a mild degree of nuclear pleomorphism. Nuclear hyperchromasia, conspicuous nucleoli, and mitotic figures were observed very rarely in the MELF area. Both the MELF and conventional tumor areas showed strong and diffuse nuclear p16 immunoreactivity (block p16 positivity; Figure 3H). The p16 expression pattern of the MELF area was identical to that of a conventional tumor area. Immunostaining for p16 highlighted the tumor cell nuclei and cytoplasm of ruptured glands and fragmented epithelial strips as well as individually scattered tumor cells.

### 3.3. Pathological Characteristics

Table 2 summarizes the pathological characteristics. All cases (10/10) were diagnosed as HPVA EACs. Nine cases were classified as usual type, and one was mucinous not otherwise specified type. Based on diffuse stromal desmoplasia and myxoinflammatory responses, all cases were classified as Silva pattern C tumors. The size of the tumor ranged 1.5–7.0 cm (median = 3.9 cm; mean = 4.1 cm). Five tumors invaded the entire stromal thickness, and four tumors involved the deep cervical stroma. The remaining tumor measured 2 mm in the deepest invasion depth. LVSI was identified in six cases, in which the number of lymphatic tumor emboli did not exceed four per slide (i.e., focal LVSI). Extension to the uterine corpus (endomyometrial extension) was noted in eight cases. The uterine serosal extension was absent. Four and two tumors involved the parametrium and vagina, respectively. None of the patients had LNM. Taken together, the initial stages were distributed as follows: IA1 (1/10), IB2 (3/10), IB3 (1/10), IIA2 (1/10), and IIB (4/10).

### 3.4. Immunostaining Results

**Figure 4:**
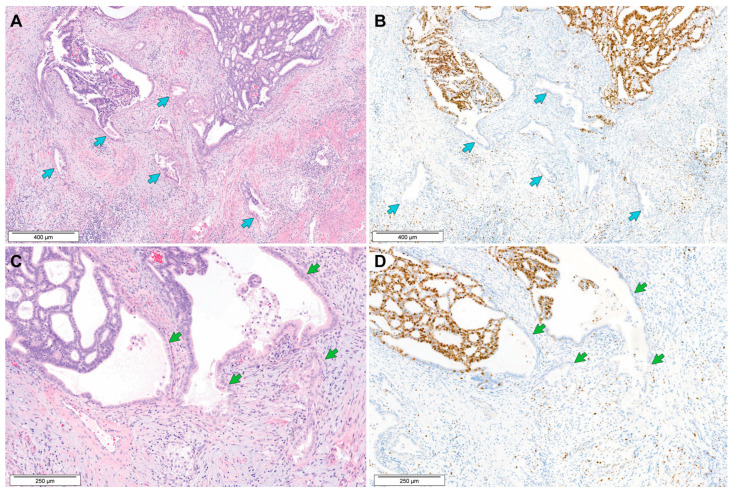
Ki-67 immunostaining results of EAC with MELF pattern. (**A**,**B**) The MELF area (blue arrows) shows reduced proliferative activity compared to the conventional invasion area. Ki-67-positive tumor cells in the MELF area obviously number fewer than in the conventional tumor area. (**C**,**D**) The lining epithelium of the MELF glands (green arrows) rarely reacted with Ki-67, whereas the conventional neoplastic glands (left upper corner) intensely and uniformly express Ki-67. Staining method: (**A**,**C**) hematoxylin and eosin staining; (**B**,**D**) immunohistochemical staining. Original magnification: (**A**,**B**) 40×; (**C**,**D**) 100×.

Table 3 summarizes the Ki-67 immunostaining results. Two cases (Cases 9 and 10), in which the tumor tissues were insufficient for additional testing, were unavailable for immunostaining. The Ki-67 highlighted the tumor cells in both the MELF and conventional tumor areas. All examined cases consistently exhibited noticeably increased proliferative activity in the conventional tumor area, with a mean Ki-67 labelling index of 76.15 ± 17.73% (range = 30.8 ± 4.66%–97.4 ± 1.95%). In contrast, in the MELF area, only a small amount of tumor cells was positive for Ki-67, with a mean Ki-67 labelling index of 16.03 ± 5.95% (range = 7.00 ± 2.00%–21.4 ± 3.05%). The Ki-67 labelling index of the MELF area was significantly lower than that of the conventional tumor area (*p* = 0.001). Figure 4 demonstrates the difference in Ki-67 expression between the MELF area and conventional tumor area.

## 4. Discussion

MELF pattern of invasion represents a unique morphology of myometrial or stromal invasion. At the invasive tumor front, stromal desmoplasia is associated with inflammatory responses, resulting in a poorly circumscribed, lobulated tumor border. Scattered irregular-shaped or ruptured neoplastic glands have a single layer of epithelial cells possessing abundant eosinophilic cytoplasm. Polygonal-shaped tumor cells resembling histiocytes form small clusters or are scattered singly in myxoinflammatory stroma. This distinctive pattern has been shown to be associated with LVSI and LNM. Stewart et al. [27] reported that approximately two-thirds of MELF-positive EEC patients had LVSI, with a significantly higher frequency compared to the MELF-negative group. Joehlin-Price et al. [11], Hertel et al. [12], and Altunpulluk et al. [28] showed that patients with MELF-positive EEC had LNM more frequently than those with MELF-negative tumors, raising the possibility that MELF pattern may be a risk factor for LNM and may serve as a predictive factor. However, it remains debatable whether MELF pattern itself is an independent predictor for LNM, due to conflicting data across studies [28,29,30,31]. Euscher et al. [29] and Song et al. [31] found that MELF pattern was not an independent predictive factor of advanced-stage disease or LNM, indicating that it needs more studies to show whether MELF pattern has an impact on the prognosis of EEC patients. Similarly, the prognostic value of MELF pattern has not been completely clarified, again due to contradictory results [8,10,32,33]. Although the association between MELF pattern and the survival of EEC patients has been widely investigated, most studies have not been able to reveal significant correlations between MELF pattern and adverse outcomes. Kihara et al. [8] reported that MELF pattern observed in low-grade EEC was significantly associated with an advanced stage and LNM, but patients with MELF-positive EEC did not show significantly worse DFS than those without MELF pattern. Similarly, Espinosa et al. [32] found no significant difference in DFS according to the presence of MELF pattern. Other researchers also have documented no significantly adverse outcomes in relation to the MELF pattern [10,33].

Regarding EAC, Segura et al. [14] conducted a multicenter study to evaluate the prognostic value of MELF pattern in a large cohort of 457 patients. The majority of MELF-positive EACs were classified as Silva pattern C tumors, and approximately two-thirds of them showed LVSI. MELF-positive EAC patients showed lower OS rates and shorter survival periods than the MELF-negative group, but again, MELF pattern did not independently predict worse survival. In agreement with the previous study, all patients included in this study had Silva pattern C EACs, and half or more of them showed LVSI and full-thickness stromal invasion. However, only two of the ten patients developed recurrences, and only one patient died of disease. Since we could not perform survival analysis, due to the small number of cases, it seems difficult to determine the value of the MELF pattern as a prognostic factor. Nevertheless, pathologists should carefully determine the presence of MELF pattern in EAC, at least because differences in the pathological characteristics of MELF-positive tumors and MELF-negative tumors exist [14]. Furthermore, additional studies on larger cohorts are necessary to establish the prognostic value of MELF pattern in EAC.

It has been reported in EEC that the tumor cells and glands representing MELF pattern are rarely positive for Ki-67, suggesting that MELF pattern is associated with cellular senescence or growth arrest [8,15,34]. Kihara et al. [8] suggested that these findings can explain why there is no significant difference in patient survival between MELF-positive and MELF-negative groups, despite more frequent LVSI and LNM in patients with EEC with MELF pattern. In line with these data, we described in a recent case study that the MELF area of EAC showed very low Ki-67 immunoreactivity compared to a conventional tumor area. In this study, we confirmed our previous observation in the single-case report that Ki-67 expression was significantly reduced in the MELF area compared to the conventional tumor area. It is worth noting that MELF pattern is associated with reduced proliferative activity, even though the characteristic morphology of MELF, such as fragmented glands, individually dispersed tumor cells, diffuse stromal desmoplasia, and intense inflammatory infiltrates, seemed to increase the tendency of invasion. Our observation of significant differences between MELF and conventional tumor areas in Ki-67 labelling should be confirmed in further investigations with a larger number of cases.

The pathogenetic mechanisms associated with MELF pattern in both EEC and EAC remain unknown. In a recent study by van den Heerik et al. [35], significantly more MELF-positive cases were found within endometrial carcinomas without catenin, beta 1 (*CTNNB1*) mutation. This association of MELF pattern with wild-type *CTNNB1* has been previously reported, and the absence of MELF pattern was even suggested as one the most sensitive predictors of *CTNNB1* mutation [36]. In another recent study by Tahara et al. [37], nicotinamide N-methyltransferase (NNMT) was found to be related to MELF pattern in EEC and promotes cell migration and invasion via the suppression of H3K9me2 methylation. They speculated that on the invasive tumor front, the elevated expression of NNMT might contribute to the characteristic morphology of MELF through the suppression of H3K9me2 followed by the enhancement of various pathways related to cell migration and invasion [37]. Meanwhile, the molecular features of MELF pattern in EAC have only been investigated in our recent case study [15]. In this single-case report, we conducted targeted sequencing analysis using tissue samples obtained from the MELF and conventional tumor areas of usual-type HPVA EAC. The mutational profiles of the MELF and conventional tumor areas were the same as each other [38,39].

As shown in Table 4, several previous studies have been conducted to compare the results of immunostaining for candidate biomarkers between conventional tumor and MELF areas. Santoro et al. [40] performed immunostaining for mismatch repair (MMR) proteins in 129 EECs. A loss of MMR protein expression was observed in 11 of the 28 MELF-positive cases and 45 of the 101 MELF-negative cases. MELF-negative cases showed a higher frequency of mutL homolog 1 (MLH1)/postmeiotic segregation increase and *Saccharomyces cerevisiae* 2 (PMS2) loss (32.7%) than MELF-positive cases (21.4%), whereas the prevalence of mutS homolog 2 (MSH2)/mutS homolog 6 (MSH6) loss was higher in MELF-positive cases (7.1% vs. 4.0%). Consistent with these findings, Song et al. [31] also observed that MELF-positive EECs showed a loss of MSH2/MSH6 expression more frequently than MELF-negative tumors, even though the difference was not statistically significant. Stewart et al. [27] evaluated the expression status of cell-cycle-regulatory proteins, including cyclin D1, p16, and β-catenin, in EECs with MELF pattern. In the conventional tumor area, cyclin D1 and p16 were frequently expressed in more than half of the tumor cells, and membranous β-catenin expression was preserved. In the MELF area, the expressions of cyclin D1 and p16 were strong but sometimes absent in the contiguous or adjacent conventional tumor area. The MELF area also displayed loss or disruption of membranous β-catenin staining. The upregulation of cyclin D1 and p16, together with the loss of membranous β-catenin expression in the MELF area, is similar to the epithelial–mesenchymal transition observed in other malignancies and suggests that MELF pattern represents an active rather than a degenerative cellular process. Similarly, Stewart et al. [41] assessed fascin expression in EEC and observed that the tumor cells in MELF pattern showed strong fascin immunoreactivity, often contrasting with the adjacent negative or more weakly stained conventional tumor area. The localized increase in fascin expression in MELF-type epithelium supports the proposal that MELF changes represent areas of active tumor invasion. They also observed that MELF pattern invasion was associated with the loss of galectin-3 expression in EEC [42]. Zinovkin et al. [43] focused on the prognostic significance of vascular endothelial growth factor (VEGF) and galectin-1 in EEC with MELF pattern. The expressions of VEGF and galectin-1 were significantly higher in the MELF-positive group. Both of these proteins were found to independently predict the OS of EEC patients, suggesting that MELF pattern, in conjunction with angiogenic markers, could serve as a prognostic factor in EEC. Tahara et al. [44] documented that the programmed cell death-ligand 1 (PD-L1) protein was strongly expressed in both the tumor cells and immune cells at the invasive front of grade 1 EEC showing MELF pattern, suggesting a possible therapeutic role for targeting programmed cell death 1/PD-L1 axis in EEC. They also reported the link between a high expression of S100A4 [45], serum deprivation-response protein (SDPR) [46], and NNMT [37] with MELF pattern. They demonstrated not only that high S100A4 expression contributes to an aggressive phenotype of EEC, but also that its elevated expression is closely related to MELF pattern. Using laser microdissection and RNA sequencing, they identified preferentially expressed genes in the MELF area. NNMT was associated with MELF pattern and invasiveness. Immunostaining revealed high NNMT expression in the invasive tumor front showing MELF pattern, suggesting the potential of NNMT inhibitors, envisioned for metabolic disorder treatment, as effective for managing EEC.

This study had some limitations. First, we enrolled patients with EAC showing MELF pattern who underwent surgery at a single institution. As this study did not include patients who were diagnosed with MELF-positive EAC at other institutions or MELF-negative EAC for comparative analysis, the cohort size was relatively small. Second, since we did not analyze the statistical differences in survival according to the presence of MELF, it seems difficult to determine the value of MELF pattern as a prognostic factor. Additional studies on larger cohorts are necessary to establish the prognostic value of MELF pattern in EAC. Third, we did not use morphometric or computer-assisted analyses for the quantification of Ki-67-positive tumor cells. Comparing the manual counting and computerized analysis of Ki-67 labelling index may better address its clinicopathological significance.

## 5. Conclusions

We demonstrated that MELF-positive EAC exhibited some adverse clinicopathological characteristics reflecting aggressive behavior. We also found that the MELF area had significantly lower proliferative activity than the conventional tumor area. Immunostaining for Ki-67 revealed that the MELF area exhibited a lower Ki-67 labelling index than the conventional tumor area in all examined cases. Despite the observation that MELF pattern is characterized by several histological features strongly indicating destructive stromal invasion, we confirmed that MELF pattern is significantly associated with low proliferative activity.

## Figures and Tables

**Table 1:** Clinical characteristics, treatment, and survival of 10 patients with EAC showing MELF pattern.

Case No.	Age (Years)	Initial Clinical Presentation	Imaging Finding	Surgical Treatment	Post-Operative Treatment	Post-Operative Recurrence	Treatment for Recurrence	DFS (Months)	Survival Status	OS (Months)
1	27	AGC on cytology	A 3.3 cm cervical cancer; no parametrial or vaginal extension; no nodal or distant metastasis	RH, BS, PLND	CCRT	Absent	None	9	Alive	9
2	46	Vaginal bleeding	A 7 cm cervical cancer with parametrial extension; no vaginal extension; no nodal or distant metastasis	RH, LSO, RS, PLND	CCRT	Absent	None	9	Alive	9
3	39	ADC on cytology	A 3.5 cm cervical cancer; no parametrial or vaginal extension; no nodal or distant metastasis	RH, BS, PLND	RT	Absent	None	13	Alive	13
4	35	Vaginal bleeding	A 4.2 cm cervical cancer; no parametrial or vaginal extension; no nodal or distant metastasis	RH, BS, PLND	RT	Absent	None	14	Alive	14
5	77	Vaginal bleeding	A 3 cm cervical cancer; no parametrial or vaginal extension; no nodal or distant metastasis	RH, BSO, PLND	RT	Absent	None	25	Alive	25
6	66	ASC-H on cytology	A 3.8 cm cervical cancer; no parametrial or vaginal extension; no nodal or distant metastasis	RH, BSO, PLND	RT	Absent	None	30	Alive	33
7	62	AGC on cytology	A 2.9 cm cancer involving the lower uterine segment with a 2.6 cm hematoma in the endometrial cavity; no cervical stromal extension; no nodal or distant metastasis	TH, BSO, PLND, PALND	None	Present (PALN)	NA *	10	NA *	NA *
8	47	ADC on cytology	A 4.5 cm cervical cancer; no parametrial or vaginal extension; suspected pelvic lymph node metastasis; no distant metastasis	RH, BSO, PLND	RT	Absent	None	73	Alive	73
9	66	HSIL on cytology	Not applicable	TH, BSO	RT	Absent	None	58	Alive	58
10	48	ADC on cytology	A 5 cm cervical cancer with endomyometrial extension and probable parametrial invasion; no vaginal extension; no nodal or distant metastasis	RH, BSO, PLND	CCRT	Present (lungs)	Surgery, chemotherapy	42	Dead	93

Abbreviations: ADC: adenocarcinoma; AGC: atypical glandular cells; ASC-H: atypical squamous cells, cannot exclude high-grade squamous intraepithelial lesion; BS: bilateral salpingectomy; BSO: bilateral salpingo-oophorectomy; CCRT: concurrent chemoradiation therapy; DFS: disease-free survival; HSIL: high-grade squamous intraepithelial lesion; LSO: left salpingo-oophorectomy; OS: overall survival; NA: not applicable; PALN: para-aortic lymph node; PALND: para-aortic lymph node; PLND: pelvic lymph node dissection; RH: radical hysterectomy; RS: right salpingectomy; RT: radiation therapy; TH: total hysterectomy. * Lost to follow-up.

**Table 2:** Pathological characteristics of 10 patients with EAC showing MELF pattern.

Case No.	Histotype	Greatest Dimension (mm)	Invasion Depth/ Stromal Thickness (mm)	Silva Pattern	LVSI	Endomyometrial Extension	Serosal Extension	Parametrial Extension	Vaginal Extension	LNM	Initial Stage
1	HPVA, usual type	33	16/16	C	Focal	Present	Absent	Present	Present	Absent (0/17)	IIB
2	HPVA, mucinous NOS type	70	9/9	C	Absent	Present	Absent	Present	Absent	Absent (0/14)	IIB
3	HPVA, usual type	35	13/13	C	Focal	Present	Absent	Absent	Absent	Absent (0/9)	IB2
4	HPVA, usual type	42	12/14	C	Absent	Absent	Absent	Absent	Present	Absent (0/23)	IIA2
5	HPVA, usual type	40	10/11	C	Absent	Present	Absent	Absent	Absent	Absent (0/10)	IB3
6	HPVA, usual type	38	11/12	C	Focal	Present	Absent	Absent	Absent	Absent (0/14)	IB2
7	HPVA, usual type	35	12/15	C	Absent	Present	Absent	Absent	Absent	Absent (0/40)	IB2
8	HPVA, usual type	45	20/20	C	Focal	Present	Absent	Present	Absent	Absent (0/12)	IIB
9	HPVA, usual type	15	2/12	C	Focal	Absent	Absent	Absent	Absent	NA	IA1
10	HPVA, usual type	55	15/15	C	Focal	Present	Absent	Present	Absent	Absent (0/27)	IIB

Abbreviations: HPVA: human papillomavirus; LNM: lymph node metastasis; LVSI: lymphovascular space invasion; NA: not applicable; NOS: not otherwise specified.

**Table 3:** Ki-67 labelling indices in eight cases of EAC showing MELF pattern.

Case No.	Ki-67 Labelling Index (%; Mean ± SD)		*p* Value
	Conventional Tumor Area	MELF Area	
1	91.40 ± 2.88	21.4 ± 3.05	0.001 *
2	89.20 ± 2.39	20.40 ± 2.30	
3	97.40 ± 1.95	21.20 ± 2.78	
4	79.40 ± 4.83	20.60 ± 4.39	
5	30.80 ± 4.66	7.00 ± 2.00	
6	74.20 ± 3.42	10.60 ± 0.93	
7	71.60 ± 4.98	14.20 ± 2.78	
8	75.20 ± 3.27	12.80 ± 3.42	
Total	76.15 ± 17.73	16.03 ± 5.95	

Abbreviations: SD: standard deviation. * Statistically significant.

**Table 4:** Summary of the previously published literature regarding potential biomarkers for EAC showing MELF pattern.

No.	Authors (Year Published)	Biomarker	Clinicopathological Significance
1	Stewart et al. (2009) [47]	Cell-cycle-regulatory proteins	Cell-cycle-regulatory proteins were heterogeneously expressed in the invasive tumor front of EEC. Particularly, the MELF area strongly expressed cyclin D1 and p16 but lost membranous β-catenin expression, suggesting that this invasion pattern represents an active rather than a degenerative cellular process.
2	Stewart et al. (2010) [42]	Galectin-3	EEC exhibited the microanatomical variation in galectin-3 expression. The MELF area showed a reduced galectin-3 expression, contrasting with the adjacent galectin-3-positive conventional tumor area. Loss of galectin-3 expression in the MELF area suggests the potential role of galectin inhibitors in treating EEC.
3	Stewart et al. (2011) [41]	Fascin	The MELF area of EEC showed strong fascin immunoreactivity, contrasting with the adjacent negative or weakly stained conventional tumor area. Fascin overexpression in the MELF area supports the notion that MELF pattern represents areas of active tumor invasion.
4	Tahara et al. (2016) [45]	S100A4	Strong and diffuse S100A4 expression was observed in the MELF area, suggesting that S100A4 overexpression, which contributes to an aggressive phenotype of EEC, is associated with MELF pattern.
5	Tahara et al. (2019) [46]	SDPR	SDPR was related to histological features associated with invasiveness, such as poor differentiation, lymphatic invasion, and MELF pattern.
6	Zinovkin et al. (2019) [43]	VEGF and galectin-1	The expression levels of VEGF and galectin-1 were significantly increased in MELF-positive EECs. MELF pattern independently predicted DFS of EEC patients, but not OS. In contrast, the expressions of VEGF and galectin-1 were an independent prognostic factor for OS.
7	Santoro et al. (2021) [40]	MMR proteins	Higher prevalence of MSH2/MSH6 loss in MELF-positive EEC and MLH1/PMS2 loss in MELF-negative EEC suggests a different molecular signature.
8	Tahara et al. (2021) [37]	NNMT	High NNMT expression was observed in the MELF area of EEC, raising the possibility that NNMT inhibitors would be effective for the treatment of EEC.
9	Tahara et al. (2022) [44]	PD-L1	In EEC cases with MELF pattern, the tumor cells expressed PD-L1 significantly higher in the invasive tumor front than in the surface area, raising the potential therapeutic role of PD-1/PD-L1 immunotherapy in treating MELF-positive EEC.
10	Song et al. (2022) [31]	MMR proteins	Consistent with the data reported by Santoro et al. [40], higher prevalence of MSH2/MSH6 loss in MELF-positive EEC and MLH1/PMS2 loss in MELF-negative EEC indicate a distinct and specific pattern of MMR-altered profile according to the presence of MELF pattern.

## Data Availability

No new data were created or analyzed in this study. Data sharing is not applicable to this article.
